# Inhibition of highly productive HIV-1 infection in T cells, primary human macrophages, microglia, and astrocytes by *Sargassum fusiforme*

**DOI:** 10.1186/1742-6405-3-15

**Published:** 2006-05-25

**Authors:** Elena E Paskaleva, Xudong Lin, Wen Li, Robin Cotter, Michael T Klein, Emily Roberge, Er K Yu, Bruce Clark, Jean-Claude Veille, Yanze Liu, David Y-W Lee, Mario Canki

**Affiliations:** 1 Center for Immunology and Microbial Disease, Albany Medical College, Albany, NY, USA; 2 Department of Microbiology and Immunology, Dartmouth Medical School, Lebanon, NH, USA; 3 Department of Ob/Gyn, Albany Medical Center, Albany, NY, USA; 4 Bio-Organic and Natural Products Laboratory, Mailman Research Center, McLean Hospital, Harvard Medical School, Belmont, MA, USA

## Abstract

**Background:**

The high rate of HIV-1 mutation and increasing resistance to currently available antiretroviral (ART) therapies highlight the need for new antiviral agents. Products derived from natural sources have been shown to inhibit HIV-1 replication during various stages of the virus life cycle, and therefore represent a potential source of novel therapeutic agents. To expand our arsenal of therapeutics against HIV-1 infection, we investigated aqueous extract from *Sargassum fusiforme *(*S. fusiforme*) for ability to inhibit HIV-1 infection in the periphery, in T cells and human macrophages, and for ability to inhibit in the central nervous system (CNS), in microglia and astrocytes.

**Results:**

*S. fusiforme *extract blocked HIV-1 infection and replication by over 90% in T cells, human macrophages and microglia, and it also inhibited pseudotyped HIV-1 (VSV/NL4-3) infection in human astrocytes by over 70%. Inhibition was mediated against both CXCR4 (X4) and CCR5 (R5)-tropic HIV-1, was dose dependant and long lasting, did not inhibit cell growth or viability, was not toxic to cells, and was comparable to inhibition by the nucleoside analogue 2', 3'-didoxycytidine (ddC). *S. fusiforme *treatment blocked direct cell-to-cell infection spread. To investigate at which point of the virus life cycle this inhibition occurs, we infected T cells and CD4-negative primary human astrocytes with HIV-1 pseudotyped with envelope glycoprotein of vesicular stomatitis virus (VSV), which bypasses the HIV receptor requirements. Infection by pseudotyped HIV-1 (VSV/NL4-3) was also inhibited in a dose dependant manner, although up to 57% less, as compared to inhibition of native NL4-3, indicating post-entry interferences.

**Conclusion:**

This is the first report demonstrating *S. fusiforme *to be a potent inhibitor of highly productive HIV-1 infection and replication in T cells, in primary human macrophages, microglia, and astrocytes. Results with VSV/NL4-3 infection, suggest inhibition of both entry and post-entry events of the virus life cycle. Absence of cytotoxicity and high viability of treated cells also suggest that *S. fusiforme *is a potential source of novel naturally occurring antiretroviral compounds that inhibit HIV-1 infection and replication at more than one site of the virus life cycle.

## Background

Macrophages and T cells are major targets for HIV-1 infection [1]. While macrophages are key cellular reservoir and a source of newly replicating HIV-1 throughout the infection, a global decline in T cell population leads to the eventual collapse of the immune system, development of clinical manifestations of AIDS, and the ultimate death of the host. Highly active antiretroviral therapy (HAART) has greatly extended the lifespan of HIV-infected individuals, however the AIDS epidemic continues to expand globally and the long-term control of HIV-1 infection remains an elusive goal. Current HAART regiments, with the exception of recent fusion inhibitor (T-20), include inhibitors of two key viral enzymes, reverse transcriptase and protease [2-4]. By using combinations of reverse transcriptase and protease inhibitors in HAART, dramatic reductions in the level of chronic HIV-1 viremia have been achieved in a majority of patients [2,4]. However, both reverse transcriptase and protease inhibitors have significant clinical side effects [5-7]. Initial optimism that the natural decay of virus-producing cells in the presence of HAART would lead to eradication of virus was short-lived [8,9]. Long-term follow-up of HAART-treated individuals revealed very slow rates of decline of HIV-1 in some individuals, with continued low-level replication of virus in macrophages and T cells, and viral persistence in several tissue compartments, such as the CNS, not readily accessible to current therapies [5,9-11]. Studies in a macaque model of simian immunodeficiency virus (SIV) viral persistence in the brain, have suggested that in individuals on HAART with suppressed viral load, the CNS may act as a long-term viral reservoir [12].

HIV-1 infected human macrophages are the primary route of virus entry into the CNS [13]. Within the CNS, active virus replication is mediated by macrophages and microglia, while astrocytes are nonproductively infected [14]. The number of astrocytes in the brain ranges up to 2 × 10^12^, and while only 1% of these cells may be latently infected, the total number of infected astrocytes contributing to neuropathology, may be substantial [15,16]. Brain macrophages, microglia, and astrocytes have been shown to be responsible for some of the neuropathologic manifestations of the HIV-associated dementia (HAD), which develops in about 20–30% of AIDS patients [14,17]. Although HAART has decreased frequency of HAD, it does not provide full protection or reversal of HAD [18]. Protease inhibitors and some of the nucleoside analogues used in HAART have poor CNS penetration, and drug resistance in this compartment has recently been reported, further underscoring need for discovery of new drugs [12,19,20].

Continued virus replication in the presence of HAART increases the likelihood and frequency of generating new multi-drug-resistant (MDR) HIV-1 strains, as demonstrated by the observation that approximately 20% of all new HIV-1 infections are with viruses resistant to the currently available drugs [21,22]. Consequently, concerted efforts towards the discovery and development of novel inhibitors of HIV-1 infection and replication must persist if continued viral repression and possibility of virus eradication are to be achieved.

We investigated a number of natural products, and identified *S. fusiforme *extract as a potent inhibitor of HIV-1 replication in T cells, in primary human macrophages, microglia, and astrocytes. While many natural products have been screened for anti-HIV activity [23,24], including sulfated polysaccharides derived from sea algae [25,26], *S. fusiforme *extract has not been investigated up until now [27].

## Results

### *S. fusiforme *does not inhibit cell growth or viability

**Figure 1:**
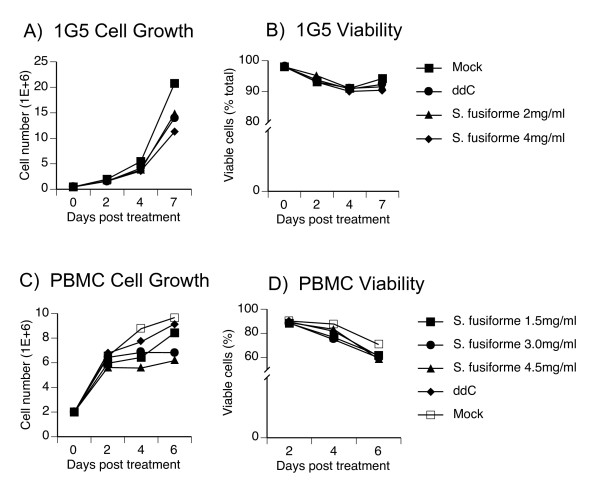
**Analysis of growth kinetics and viability in T cells treated with *S. fusiforme***. 1G5 T cells were treated with 2 mg/ml or 4 mg/ml *S. fusiforme*, or with 10^-6 ^M ddC, or were mock treated. (A) Total cell number, and (B) % viable cells from total, was monitored at the indicated time points after infection, by trypan blue exclusion assay by counting at least 200 cells each in three different fields under ×20 magnification using an Olympus BH-2 fluorescence microscope. Experiment was repeated with primary human PBMC's treated with 1.5, 3, or 4.5 mg/ml *S. fusiforme*, or with 10^-6 ^M ddC, or mock treated, and measured (C) Total cell number, and (D) % viable cells from total. PBMC's experiments are representative of 3 separate experiments, with SEM less than 5% (not shown).

To establish a non-toxic working concentration, we tested for cell growth and viability kinetics in response to treatment with *S. fusiforme *whole aqueous extract. T cells were treated with either 2 or 4 mg/ml *S. fusiforme*, 10^-6 ^M ddC, or were mock treated (Fig 1). In 1G5 cells, growth kinetics remained similar, except for the highest 4 mg/ml treatment on day 7 that decreased cell growth by 19% compared to ddC treatment, indicating possible toxicity at this dose (Fig 1A). In parallel we also measured cell viability by trypan blue exclusion assay. Regardless of treatment, cell viability remained above 90%, which was comparable to mock treated cultures (Fig. 1B). We repeated this experiment with HIV-1 infected 1G5 cells, with similar results (not shown). Because of toxicity relevance in primary human cells, we also measured cell growth and viability in human peripheral blood mononuclear cells (PBMC), with similar results (Fig 1C and 1D). Cells treated with either 3 or 4.5 mg/ml *S. fusiforme *exhibited somewhat slower growth kinetics on day 6 after treatment, as compared to 1.5 mg/ml *S. fusiforme*, ddC or mock treated cells (Fig 1C). However, viability of *S. fusiforme *and ddC treated cells remained similar through day 6 of follow-up, with the overall PBMC's viability declining over time, as compared to 1G5 T cell line (compare Fig. 1D to 1B ).

Based on these results we conclude that treatment with less than 4 mg/ml *S. fusiforme *extract, does not inhibit cell growth, is not toxic to cells, and is suitable for *in vitro *testing of HIV-1 inhibition in 1G5 cells.

### *S. fusiforme *inhibits HIV-1 infection in T cells in a dose dependant manner

**Figure 2:**
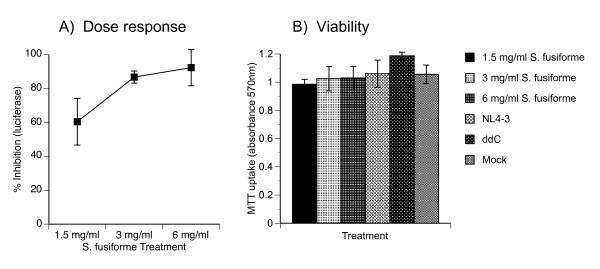
**Dose response of HIV-1 inhibition and cell viability in T cells treated with *S. fusiforme***. 1G5 T cells were treated for 24 h with increasing concentrations of *S. fusiforme*, or with 10^-6 ^M ddC, as indicated; then infected with CXCR4 tropic HIV-1 (NL4-3) at multiplicity of infection (moi) of 0.01 for 1.5 h, washed 3 times, and returned to culture with same concentrations of each treatment for the duration of the experiment. (A) On day 3 after infection, intracellular luciferase gene marker expression was measured from cell lysates adjusted to same number of viable cells by MTT. Percent inhibition of HIV-1 was calculated utilizing formula in the Methods section, and plotted on the Y-axis as % Inhibition. In parallel, (B) cell viability for each treatment was quantified by MTT uptake, measured at 570 nm absorbance. Data are mean +/- SD of triplicates. Representative of three separate experiments.

Next, we investigated *S. fusiforme *ability to inhibit HIV-1 infection in T cells. We chose 1G5 T cells, which are stably transfected with HIV-LTR-luciferase gene construct, have low basal level of luciferase expression and are sensitive to HIV-1 *tat *activation, which makes them a useful tool for testing HIV-1 inhibitors [28]. Cells were treated with increasing concentrations of *S. fusiforme *extract and infected with NL4-3. On day 3 after infection, equal numbers of viable cells were analyzed for intracellular luciferase expression, and cell viability was measured by MTT uptake assay (Fig. 2). Percent HIV-1 inhibition was calculated by comparison to control infected untreated cell cultures, which expressed 18,797 relative light units (RLU) of luciferase (not shown). Treatment with 1.5, 3, and 6 mg/ml of *S. fusiforme *extract inhibited HIV-1 replication in a dose dependant manner, by 60.4, 86.7, and 92.3%, respectively (Fig. 2A). As expected, treatment with positive control HIV-1 reverse transcriptase (RT) inhibitor ddC, blocked virus replication by over 98% (not shown). In parallel, we tested for the MTT uptake by viable cells, which remained high regardless of *S. fusiforme *treatment, and was similar to ddC, as well as to viability of mock treated cells (Fig. 2B).

Based on these results we conclude that *S. fusiforme *treatment inhibits HIV-1 replication in T cells in a dose dependant manner, inhibition is similar to that achieved with ddC treatment, and treatment is not toxic to cells.

### *S. fusiforme *inhibition is non-toxic and can be sustained over extended periods

**Figure 3:**
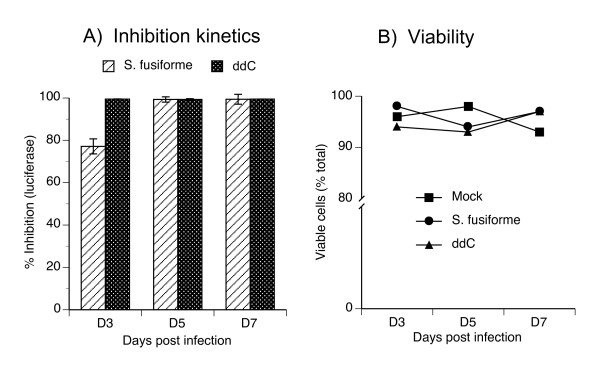
**Time course of HIV-1 inhibition and viability in T cells**. 1G5 T cells were 24 h treated with either 2 mg/ml *S. fusiforme*, or with 10^-6 ^M ddC; then infected with NL4-3 at 0.01 moi for 1.5 h, washed 3 times, and returned to culture with same concentration of each treatment for the duration of the experiment. On day 3 post-infection, (A) gene expression of intracellular luciferase was measured from cell lysates adjusted to same number of viable cells, and % inhibition calculated and plotted on the Y-axis. Data are mean +/- SD of triplicates. In parallel, (B) cell viability was determined by trypan blue exclusion assay by counting at least 200 cells each, in three different fields under ×20 magnification using an Olympus BH-2 fluorescence microscope.

Next, we tested for the duration of HIV-1 inhibition in 1G5 T cells, treated with either 2 mg/ml *S. fusiforme *or with 10^-6 ^M ddC. Infection was monitored by luciferase expression from cells equalized to same number of viable cells by MTT assay, at the indicated time points after infection (Fig. 3A). HIV-1 infection in untreated cells gradually increased from 16,110 RLU expressed on day 3, to 86,720 RLU on day 7 after infection, which demonstrated highly productive and *de novo *HIV-1 synthesis (not shown). Treatment with 2 mg/ml *S. fusiforme *inhibited this infection by 77, 99, and 99% on day 3, 5, and 7, respectively (Fig. 3A). As expected, inhibition by ddC was 99% at each time point tested. Based on these results we calculated IC50 to be 0.86 mg. Similar time course inhibition results were obtained in CEM T cells (not shown).

In parallel to infection kinetics, we also tested cell viability by trypan blue exclusion assay (Fig. 3B). Cell viability in *S. fusiforme *treated cultures remained high at 98, 94, and 97% viable cells on day 3, 5, and 7, respectively. Cell viability in ddC treated cultures was similar, and measured 94, 93, and 97% viable cells on day 3, 5, and 7, which was similar to mock treated cultures. This data confirm MTT viability results, which were used to equalize cells to same numbers of viable cells (not shown).

Collectively, these findings demonstrate that *S. fusiforme *inhibits infection and *de novo *HIV-1 synthesis, through day 7 of follow-up, and this treatment does not affect cell viability.

### *S. fusiforme *blocks HIV-1 transmission by direct cell-to-cell mechanisms of infection

**Figure 4:**
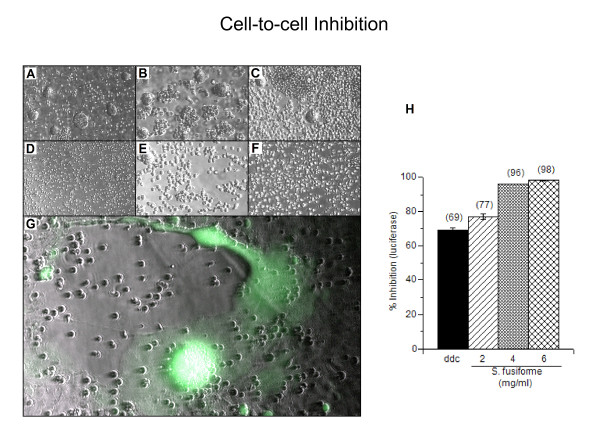
**Inhibition of cell-to-cell infection and syncytia formation**. Uninfected 1G5 T cells were pretreated for 24 h with either (A) mock, (B) 10^-6 ^M ddC, or with ddC and (B) 2 mg/ml or (C) 4 mg/ml *S. fusiforme*, or with *S. fusiforme *only at (D) 2 mg/ml or (E) 4 mg/ml. 1G5 cells were cocultivated at 1:1 ratio with CEM cells that were infected with NL4-3 at 0.01 moi. 24 h after cocultivation, cells were examined for syncytium formation using Leica DM IL Fluo microscope, ×20 magnification (A-F). Cell cultures were monitored for luciferase expression, and % inhibition was calculated from maximal luciferase expression from untreated 1G5 cells (1.9 × 10^5 ^RLU, not shown), which was plotted and is indicated on top of each bar (H). Data are mean +/- SD of triplicates. Uninfected adherent GHOST [29] cells were ddC treated and cocultivated at 1:1 ratio with HIV infected 1G5 cells for 24 h, and examined for syncytia formation by green fluorescence (G). Image shows fluorescence micrograph taken of a green fluorescent giant cell, which was superimposed on the same field phase contrast black and white image.

HIV-1 infection is spread either by free viral particles, or 100 times more efficiently by direct cell-to-cell fusion [1]. Considering that *S. fusiforme *inhibits HIV-1 infection in T cells (Fig. 3), we wanted to determine its ability to block cell-to-cell mediated viral transfer. To test this, we performed two separate experiments with different cell types (Fig 4). First, we examined the ability of HIV infected CEM cells to fuse and spread infection to uninfected 1G5 cells that were either mock treated, treated with 10^-6 ^M ddC only, or treated with increasing concentrations of *S. fusiforme *and ddC, or with *S. fusiforme *only. Pretreatment of 1G5 cells with 10^-6 ^M ddC inhibits virus replication, and therefore serves as a control for false positive luciferase readings from free virus particle infection and replication, however it does not prevent spread of infection by cell-to-cell fusion. CEM and 1G5 cells were cocultivated for 24 h at a ratio of 1:1, and examined for cell-to-cell fusion and syncytia formation by phase contrast microcopy (A-F) or by luciferase expression (H). As expected, many large syncytia were observed in co-cultures with mock treated or only ddC treated 1G5 cells (A and B). However, 1G5 treatment with 2 mg *S. fusiforme*, with or without ddC, greatly reduced cell-to cell fusion and syncytia formation (C and E). No giant cells were detected in 1G5 cells treated with either 4 mg/ml (D and F) or with 6 mg/ml (not shown) *S. fusiforme*, with or without addition of ddC. Inhibition of viral infection by cell-to-cell fusion was also confirmed by decreased luciferase expression in *S. fusiforme *treated 1G5 cells that were cocultivated with HIV infected CEM cells (H). CEM cells do not have the HIV-LTR-luciferase gene, as 1G5 cells do, and therefore luciferase readings from cocultivated cell cultures can only arise from 1G5 cells that fused and formed giant cells with infected CEM cells. 24 h after cocultivation with untreated 1G5 cells, luciferase expression measured 1.9 × 10^5 ^RLU, which represented maximal luciferase expression in the absence of any treatment (not shown). 1G5 treatment with 10^-6 ^M ddC and 2, 4, or 6 mg *S. fusiforme *inhibited cell-to-cell fusion, as measured by luciferase expression in 1G5 cells, by 77, 96, and 98%, respectively (H). Inhibition was similar in cells treated with *S. fusiforme *only, in the absence of ddC, demonstrating low rate of infection by free virus, during the 24 hours of cocultivation (not shown). In comparison, 1G5 cell treatment with only 10^-6 ^M ddC, inhibited luciferase expression by 69%.

In the second experiment, we cocultivated HIV infected and untreated 1G5 cells with uninfected and treated HIV-LTR-GFP-expressing GHOST adherent cells [29], and monitored for cell-to-cell fusion by GFP expression from GHOST cells (G). After cocultivation with infected 1G5 cells, mock or only ddC treated GHOST cells can fuse, and form syncytia that emit green florescence, which was detected by phase fluorescence microscopy. GHOST cells that were ddC treated and cocultivated with HIV-1 infected 1G5 cells, resulted in cell-to-cell fusion and fluorescent giant cell formation as is shown by fluorescence micrograph superimposed on the phase contrast black and white image of the same field (G). However, as in CEM-1G5 cocultivation experiment, no giant cells emitting green fluorescence were detected in 1G5 cells cocultivated with GHOST cells that were treated with *S. fusiforme*, with or without ddC (not shown).

Based on the results of these two different experiments, we conclude that *S. fusiforme *blocks HIV-1 infection by cell-to-cell fusion mechanism, which also prevents subsequent multinucleated cell formation and its associated cytophatic effects.

### *S. fusiforme *inhibits HIV-1 infection in primary human macrophages and brain microglia

**Figure 5:**
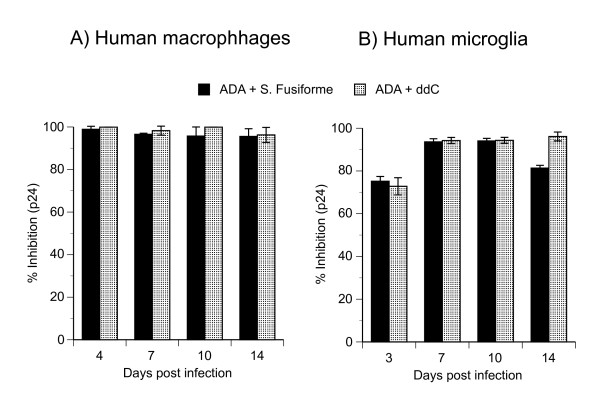
**Inhibition of HIV-1 expression in human macrophages and microglia**. Either, (A) human macrophages or (B) human fetal microglia were 24 h treated with 1 mg/ml *S. fusiforme*, or with 10^-6 ^M ddC, infected with primary CCR5-tropic isolate ADA at 0.2 pg of p24/cell for 2 h, washed 3 times, and returned to culture with same concentration of each treatment for the duration of the experiment. At the indicated time points after infection HIV-1 expression was monitored by p24 production in cell-free supernatants by ELISA, % inhibition calculated as described in Methods and plotted on the y-axis. Data are mean +/- SD of triplicates. Representative of 2 experiments.

Macrophages and brain microglia are productively infected with R5-tropic HIV-1, and are considered to be the primary source of virus replication in the periphery and in the CNS [1]. Because of their importance to HIV infection, we investigated ability of *S. fusiforme *extract to inhibit virus infection in these cells. Primary human macrophages or microglial cell cultures were treated with 1 mg/ml *S. fusiforme *extract and infected with primary R5 isolate ADA [30]. Infection was monitored by measuring viral p24 concentrations in cell-free supernatants, at the indicated time points after infection (Fig. 5).

In infected and untreated macrophage cell cultures, virus levels steadily increased from 19,097 pg of p24/ml on day 4, to a peak of infection on day 14, measuring 163,740 pg of p24/ml, indicating productive HIV-1 infection and *de novo *virus synthesis (not shown). However, treatment with 1 mg/ml *S. fusiforme *extract inhibited ADA replication (dark bars) by over 90% through day 14 after infection, which was comparable to the inhibition with ddC treatment (Fig. 5A).

Next, we treated fetal microglial cell cultures with either 1 mg/ml *S. fusiforme*, or 10^-6 ^M ddC, or mock treated, and monitored infection kinetics by p24 production in cell-free supernatants at the indicated time points after infection (Fig. 5B). As in T cells and macrophages, infected and mock treated microglia were productively infected as demonstrated by steadily increasing p24 production that reached a peak on day 14 with 2,313 pg of p24/ml (not shown). Treatment with *S. fusiforme *inhibited this infection by 75% on day 3, by over 90% on day 7 and 10, and by 81% on day 14 after infection. By comparison, virus inhibition by ddC was 72% on day 3, and thereafter remained above 90%.

In parallel to infection kinetics, we monitored cell viability by MTT assay, which remained high and was similar to uninfected cell cultures (not shown). Based on these results we conclude that *S. fusiforme *is a potent inhibitor of R5-tropic HIV-1 infection in primary human macrophages and microglia: inhibition is long lasting, not toxic to cells, and with similar inhibition kinetics to those observed in T cells (Fig. 3A).

### *S. fusiforme *inhibits HIV-1 infection during entry and post-entry events of virus life cycle

Collectively, our results demonstrate that S. fusiforme extract robustly inhibits HIV-1 infection in a number of cell types, and in a number of infection scenarios. In order to determine how this inhibition works, we tested whether the extract could block infection at a post-entry level of virus replication.

**Figure 6:**
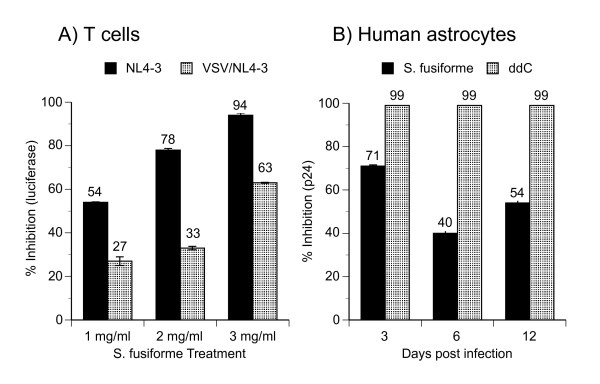
**Inhibition of infection with pseudotyped HIV-1 in T cells andhuman astrocytes**. (A) 1G5 T cells were treated with increasing concentrations of *S. fusiforme *and infected with either NL4-3 at 0.01 moi or with VSV/NL4-3 at 0.005 moi. 3 days after infection, % inhibition was calculated from luciferase expression from cell lysates adjusted to same number of viable cells by MTT. (B) Human fetal CD4 negative astrocytes were treated with 1 mg/ml *S. fusiforme*, or with 10^-6 ^M ddC, infected with VSV/NL4-3 at 0.4 moi, and infection kinetics monitored by p24 expression in cell free supernatants at the indicated time points post infection. Data are mean +/- SD of triplicates. Representative of 2 experiments.

HIV-1 pseudotyped with the vesicular stomatitis virus G-protein (VSV-G) can infect cells without interacting with CD4 and co-receptors. We extended HIV-1 tropism by pseudotyping native HIV-1 (NL4-3) with VSV-G envelope (VSV/NL4-3), which produced native NL4-3 with heterologous envelope glycoproteins that bind to commonly expressed cellular receptors. VSV/NL4-3 virus gains access to the cytoplasm by fusing out of endocytic vesicles [31]. Therefore, any block to VSV/NL4-3 replication would suggest post-entry inhibition. We treated T cells with increasing doses of *S. fusiforme*, infected with NL4-3 or VSV/NL4-3, and monitored infection by luciferase gene expression on day 3 after infection (Fig. 6A). To our surprise, *S. fusiforme *mediated dose dependant inhibition of VSV/NL4-3, inhibiting at 26.6, 32.8, and 62.6% that corresponded to 1, 2, and 3 mg/ml *S. fusiforme *extract treatment, respectively (Fig. 6A, light bars). However, overall inhibition of pseudotyped virus was markedly lower as compared to inhibition of native NL4-3, which was inhibited by 53, 78, and 93% (dark bars). Considering that pseudotyped VSV/NL4-3 has no cell surface entry restrictions, these data suggest that: 1) *S. fusiforme *blocks at a post-entry step of viral replication, and 2) inhibition is also mediated during entry process, as suggested by difference in the levels of inhibition between native NL4-3 and VSV/NL4-3 infections.

To confirm and extend the finding of post-entry inhibition in T cells, we tested for inhibition of VSV/NL4-3 in CD4-negative primary cells. Human astrocytes are CD4-negative cells that are nonproductively infected by HIV-1 *in vivo *[16], and *in vitro *[32-34]. However, we showed that, *in vitro*, these cells fully support productive virus replication after entry restriction has been bypassed [35]. Infection with VSV/NL4-3 productively infects majority of astrocytes, and serves as model system to study HIV-1 replication in these cells [35]. We infected primary human astrocytes with VSV/NL4-3, and monitored infection kinetics at the indicated time points after infection, by measuring p24 production in cell-free culture supernatants (Fig. 6B). Peak of infection was reached on day 12 with 71,000 pg of p24/ml produced in the infected and untreated cell culture, indicating ongoing virus replication (data not shown). Consistent with post-entry inhibition observed in T cells (Fig. 6A), treatment with 1 mg/ml *S. fusiforme *extract also inhibited post-entry virus replication in primary human astrocytes, by 71, 40, and 54%, on day 3, 6, and 12, respectively (Fig. 6B).

These data support our hypothesis that in addition to inhibiting viral entry, *S. fusiforme *extract also blocks viral replication during a post-entry event of the virus life cycle. However, the exact mechanisms of either entry or post-entry inhibition need to be further investigated.

## Discussion

The high rate of HIV-1 mutation and increasing resistance to currently available antiretroviral therapies underscores the need for new antiviral agents. The AIDS pandemic has been especially devastating in the Third world countries that can least afford or have easy access to current therapies, demonstrating a need for affordable treatments aimed at preventing HIV infection [36]. To expand search for novel inhibitors of HIV infection and replication, we studied and identified naturally occurring *S. fusiforme *extract as an efficient inhibitor of HIV-1 replication in T cells, in primary human macrophages, microglia, and astrocytes.

First, we demonstrated that *S. fusiforme *aqueous extract does not inhibit cell growth, is not toxic to cells, and was therefore suitable for further *in vitro *studies of HIV-1 inhibition (Fig. 1). Because it may be easier to block inefficient low level virus replication, we ensured that the observed inhibition was mediated against productive and *de novo *viral synthesis, by monitoring virus replication by either cell free p24 production or intracellular luciferase reporter gene expression. In T cells, *S. fusiforme *extract inhibited HIV-1 replication up to 90%, in a dose dependant manner (Fig. 2). This inhibition was long lasting, up to 7 days of follow-up, and was similar to the levels of inhibition observed with ddC treatment (Fig. 3).

*In vivo*, one mechanism of HIV-1 infection and viral spread is by a direct cell-to-cell fusion, between infected and uninfected cell [37]. To investigate possible inhibition of this mechanism of infection, in two separate experiments with different cell types, we cocultivated *S. fusiforme *treated cells, with HIV infected cells, and monitored for syncytia formation by microscopy, and for viral replication by luciferase expression (Fig 4). In both experiments, treatment with *S. fusiforme*, with or without ddC control for free virus infection, prevented cell-to-cell fusion and inhibited infection, in a dose dependant manner. These results demonstrate ability of *S. fusiforme *to inhibit physiologically relevant mechanism of spreading infection.

Infected macrophages act as a bridge between the periphery and the CNS, by spreading HIV-1 infection to microglia and astrocytes in the CNS [14]. Treatment with 1 mg/ml *S. fusiforme *extract inhibited active R5-tropic virus replication by 90%, in primary human macrophages and microglial cell cultures (Fig 5). In primary human astrocytes, *S. fusiforme *ihibited VSV/NL4-3 entry independent infection by 71%, which also suggested post entry inhibition of virus replication in these cells (Fig 6B). *S. fusiforme *did not inhibit cell growth or viability in these cells, which was consistent with results in T cells (Fig 1 and 2). These results demonstrate ability of *S. fusiforme *extract to inhibit HIV-1 replication in the two relevant cell types in the CNS, microglia and astrocytes. In this context, it would be of interest to determine whether *S. fusiforme *is capable of crossing the blood-brain barrier (BBB), and be an effective treatment in this important viral reservoir.

Because it was not clear which step of the virus life cycle *S. fusiforme *blocks, we investigated possibility of post entry inhibition. We tested for inhibition of infection with VSV-G pseudotyped HIV-1, which has been used to bypasses any entry restrictions [31,35]. Treatment with increasing doses of *S. fusiforme *inhibited VSV/NL4-3 infection in T cells in a dose dependant manner (Fig. 6A). However, compared to inhibition of native HIV-1, inhibition of VSV/NL4-3 was markedly lower, up to 57% lower, indicating interference with post entry steps of virus life cycle. We extended this finding by infecting CD4-negative human astrocytes with VSV/NL4-3, which was also inhibited by 71% (Fig 6B). Consistent with lower post entry inhibition in T cells, post entry inhibition in astrocytes was also lower, as compared to 99% inhibition with ddC treatment. The reasons for these inhibition differences are not clear, but given that native NL4-3 has entry restrictions and pseudotyped VSV/NL4-3 does not, we interpret these results to mean that *S. fusiforme *mediates HIV-1 inhibition during both entry and post entry steps of virus life cycle. However, the exact mechanisms of this inhibition need to be investigated. Considering *S. fusiforme *inhibition in different cell types, and with different mechanisms of action, we further postulate that this complex aqueous mixture contains more than one biologically active molecule mediating the observed HIV-1 inhibition.

## Conclusion

*S. fusiforme *extract is a potent inhibitor of HIV-1 infection in T cells, in human macrophages, microglia, and astrocytes. Inhibition is mediated during both entry and post entry events of the virus life cycle. Based on these results we propose that *S. fusiforme *is a lead candidate for bioactivity guided isolation and identification of active compounds mediating the observed HIV-1 inhibition. Identification of these compounds will allow investigation of the precise mechanisms of inhibition as well as standardization of the whole extract for potential *in vivo *use, and for development of novel antiretroviral drugs and microbicides.

## Methods

### Generation of aqueous extract from *S. fusiforme *plant material

Dried *S. fusiforme *was obtained from the wholesale distributor, South Project LTD. Hong Kong, China. To confirm content and consistency, each separate shipment was first identified botanically, and then incubated at 55°C for 6 hours to eliminate any residual moisture. The dried material was briefly washed in cold water to remove any debris or loose particulate matter, weighed and resuspended to 100 mg/ml H_2_0 in covered sterile glass beakers, and boiled at 100°C for one hour. Hot water extracts were allowed to cool to room temperature, then filtered three times through a Whatman filter paper #2, and autoclaved for 20 minutes. Each preparation was centrifuged at 100,000 × g for 1 h to remove any additional particulate matter, aliquoted and stored at -20°C until use.

### Cells and culture treatments

#### T cells

1G5 and CEM T cells were obtained from the NIH AIDS Reagent Repository and cultured in RPMI 1640 suplemented with 10% fetal bovine serum (FBS, HyClone) and penicillin-streptomycin (pen/strep).

#### Monocyte-derived human macrophages

Monocytes were recovered from peripheral blood mononuclear cells (PBMCs) by countercurrent centrifugal elutriation as previously described [30]. Monocytes were cultured as adherent monolayers (1 × 10^6 ^cells/well in 24-well plates), differentiated for 7 days in Dulbecco's modified Eagle's medium (DMEM) supplemented with macrophage colony stimulating factor (M-CSF, a generous gift from Wyeth, Cambridge, MA). Confluent cultures of fully differentiated macrophages were infected with HIV-1 CCR5-tropic ADA primary isolate, as indicated in Figure legends.

#### Isolation and culture of fetal microglial cells

Fetal microglial cells were isolated from second-trimester (gestational age, 17–19 weeks) human fetal brain tissue obtained from elective abortions in full compliance with National Institutes of Health (NIH) guidelines, as previously described [30]. Briefly, the tissue was washed with cold Hanks Balanced Salt Solution (HBSS, MediaTech), then mechanically dissociated and digested with 0.25% trypsin (Gibco) for 30 minutes at 37°C; trypsin was neutralized with FBS (HyClone). Single cell suspensions were plated in DMEM supplemented with 10% FBS, 1000 U/ml M-CSF, and pen/strep. The mixed cultures were maintained at 37°C for 7 days and the media was fully exchanged to remove any cellular debris. The microglial cells, released upon further incubation, were collected and purified by preferential adhesion. Microglia were cultured as adherent monolayers at a density of 0.1 × 10^6 ^cells/well in 24-well plates, and were infected as described in Figure legends.

#### Human fetal astrocytes

Fetal astrocytes were isolated from second-trimester (gestational age, 17–19 weeks) human fetal brains obtained from elective abortions in full compliance with National Institutes of Health (NIH) guidelines, as previously described [35]. Briefly, highly homogenous preparations of astrocytes were obtained using high-density culture conditions in the absence of growth factors in F12 Dulbecco's modified Eagle's medium (GIBCO-BRL, Gaithersburg, Md.) containing 10% FBS, pen/strep, and gentamycin. Cultures were regularly monitored for expression of the astrocytic marker glial fibrillary acidic protein (GFAP) and either HAM56 or CD68 to identify cells of monocyte/macrophage lineage. Only cultures that contained 99% GFAP-positive cells and rare or no detectable HAM56- or CD68-positive cells were used in our experiments [35].

#### Cell culture treatments

Before infection, cells were grown for 24 h in culture media with the indicated concentration of either *S. fusiforme *extract, or with 10^-6 ^M ddC, washed 3 times with HBSS (GIBCO-BRL), and infected as indicated. After infection cells were washed 3 times, and returned to culture with same concentration of each treatment for the duration of experiment.

### HIV-1 molecular clones, envelope expression vectors, and generation of pseudotyped HIV-1

T cell tropic HIV-1 molecular clone NL4-3 expresses all known HIV-1 proteins [38], and it was used to infect T cell experimental systems. VSV/NL4-3 viral stocks were prepared by cotransfection of intact NL4-3 DNA (pNL4-3) and VSV envelope expression vector (pL-VSV-G). The VSV-G expression vector pL-VSV-G was obtained from M. Emerman; it contains a VSV-G insert in the pcDNA expression vector modified by replacing the cytomegalovirus promoter with the HIV-1 long terminal repeat [39]. High-titer virus stocks, including pseudotyped virus, were produced in early passage 293T human embryonic kidney cells transfected with the respective DNA by calcium phosphate precipitation [40], as previously described [35]. Cell-free viral stocks were tested for HIV-1 p24 core antigen content by enzyme-linked immunosorbent assay (ELISA) using HIV-1 Ag kit as specified by the manufacturer (Coulter, Hialeah, Fla). Titers of infectious virus were determined by multinuclear activation of β-galactosidase indicator (MAGI) assay [41]. In our hands, a multiplicity of infection of 1 for CD4-positive T cells is equivalent to approximately 1 pg of viral p24 per cell [35]. Macrophage and microglial cells infections were performed using the HIV-1 CCR5-tropic primary isolate ADA, as previously described [30].

### Infections and analysis of HIV-1 expression by luciferase gene expression and by p24 ELISA

T cells, confluent cultures of macrophages, microglial cells, or human fetal astrocytes were infected with native or pseudotyped HIV-1 at the multiplicity of infection (moi) as indicated in the Figure legends, and were washed three times with HBSS (GIBCO-BRL) before being returned to culture. At the indicated times after infection, equal number of viable cells normalized by CellTiter 96 Non-Radioactive Cell proliferation Assay [(3-(4,5-Dimethyl-2-thiazolyl)-2,5-dephenyltetrazolium (MTT)] kit, or by trypan blue exclusion assay, were tested for luciferase expression using Luciferase Assay System kit (Promega) as specified by the manufacturer. Cell-free supernatants were tested for HIV-1 p24 core antigen content by ELISA using the HIV-1 Ag kit as specified by the manufacturer (Coulter, Hialeah, Fla).

Infected T cell cultures were analyzed for syncytium formation at the indicated time points after infection by visualizing cells under an Olympus BH-2 fluorescence microscope, and at least 4 separate wells from a 12-well plate (Costar), from identical experimental systems were analyzed.

### Calculation of percent inhibition of infection

Percent (%) inhibition was determined from either luciferase expression or p24 content, utilizing the following formula:



## Competing interests

The author(s) declare that they have no competing interests.

## Authors' contributions

MC, EEP, and DYWL participated in the design of experiments.

MC, EEP, XL, and DYWL participated in the interpretation of the results.

MC and EEP prepared the manuscript.

EEP, XL, WL, ER, RC, EKY, WBC, JCV, and YL performed the experiments.
